# Flexible anode materials for lithium-ion batteries derived from waste biomass-based carbon nanofibers: I. Effect of carbonization temperature

**DOI:** 10.1039/c7ra13639k

**Published:** 2018-02-14

**Authors:** Lei Tao, Yuanbo Huang, Xiaoqin Yang, Yunwu Zheng, Can Liu, Mingwei Di, Zhifeng Zheng

**Affiliations:** Yunnan Provincial International Joint Research Center for Bioenergy, Yunnan Provincial Engineering Laboratory for Highly-Efficient Utilization of Biomass, Yunnan Provincial University Key Laboratory for Biomass Chemical Refinery & Synthesis, College of Materials Science & Engineering, Southwest Forestry University Kunming 650224 China zhengzhifeng666@163.com youthshow@163.com +86 13700641767 +86 18787044383; College of Materials Science and Engineering, Northeast Forestry University Harbin 150040 China dimingwei@nefu.edu.cn +86 13946050478; Fujian Engineering and Research Center of Clean and High-valued Technologies for Biomass, College of Energy, Xiamen University Xiamen 361102 China

## Abstract

Carbon nanofibers (CNFs) with excellent electrochemical performance represent a novel class of carbon nanostructures for boosting electrochemical applications, especially sustainable electrochemical energy conversion and storage applications. This work builds on an earlier study where the CNFs were prepared from a waste biomass (walnut shells) using a relatively simple procedure of liquefying the biomass, and electrospinning and carbonizing the fibrils. We further improved the mass ratio of the liquefying process and investigated the effects of the high temperature carbonization process at 1000, 1500 and 2000 °C, and comprehensively characterized the morphology, structural properties, and specific surface area of walnut shell-derived CNFs; and their electrochemical performance was also investigated as electrode materials in Li-ion batteries. Results demonstrated that the CNF anode obtained at 1000 °C exhibits a high specific capacity up to 271.7 mA h g^−1^ at 30 mA g^−1^, good rate capacity (131.3 and 102.2 mA h g^−1^ at 1 A g^−1^ and 2 A g^−1^, respectively), and excellent cycling performance (above 200 mA h g^−1^ specific capacity without any capacity decay after 200 cycles at 100 mA g^−1^). The present work demonstrates the great potential for converting low-cost biomass to high-value carbon materials for applications in energy storage.

## Abstract 2

Carbon nanofibers (CNFs) with excellent electrochemical performance represent a novel class of carbon nanostructures for boosting electrochemical applications, especially sustainable electrochemical energy conversion and storage applications.

## Graphical abstract

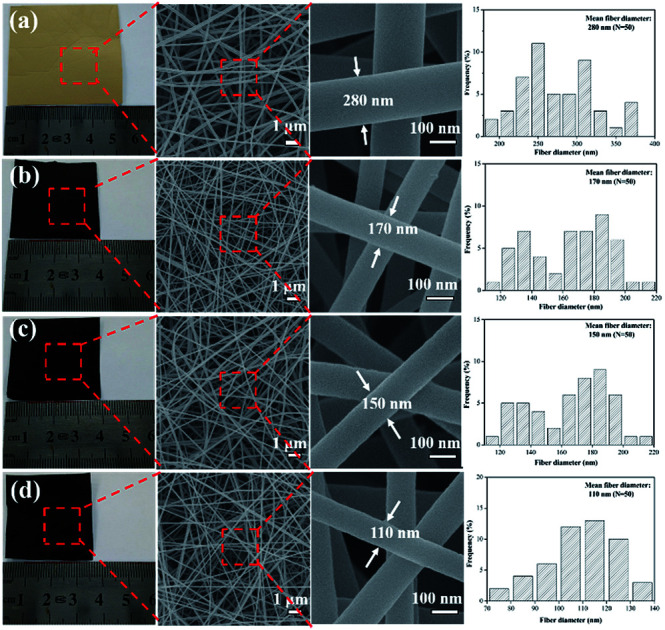

## 1 Introduction

With the severe shortage of crude oil resources and ever-growing energy consumption, there has been an increasing and urgent demand for exploring cost-effective, green and sustainable energy storage systems.^1^ Lithium ion batteries (LIBs) have attracted more and more attention as the most efficient, emerging energy storage devices that are also environmentally friendly and are being extensively applied in various applications, including various types of electric vehicles and advanced portable electronics, due to their unique features like high output voltage, long cycle life and great safety performance.^2–4^ For LIBs, the electrolyte and electrode materials are critical components, which mainly determine the overall electrochemical performance.^5^ It is well-known that carbon-based materials have been holding a predominant position and are widely employed for electrode materials, due to their low-cost, high electronic conductivity, excellent cycle stability, and wide operating voltage from 0 to 3 V.^6,7^ However, they cannot meet the ever-increasing needs and requirements for high-performance energy conversion and storage applications, due to their low specific capacity of 372 mA h g^−1^ and poor energy density (1–10 W h kg^−1^).^8,9^ Therefore, other types of carbon materials are widely studied as candidates to improve the energy density and long cycle performance in LIBs.^10^

The newly developed carbon nanomaterials, such as carbon nanofibers (CNFs),^11^ carbon nanotubes (CNTs),^12^ graphene,^13,14^ mesoporous carbons spheres,^15^ and even some carbon nanocomposites,^16,17^ have been explored to replace the traditional carbon materials for electrode materials due to their excellent electronic conductivity and abundant porosity. But the complicated and cumbersome processes restrained their extensive applications. As an example, the synthesis of template-assisted CNTs or mesoporous carbons include the fabrication of the template prior to the growth of the CNTs/mesoporous carbons, template removal after the growth, and further purification of the obtained materials. So the processing parameters in each step may influence the quality of the final product.^18^ In order to avoid such tedious and complicated process, electrospinning as a simple and convenient method could be easily to generate nanofibers from stable solution, and its carbonized products could directly act as electrode materials for LIBs, simplifying the preparation process of electrode nanomaterials.^19^ For example, Kim and co-workers prepared CNFs from polyacrylonitrile (PAN) and applied them as electrode materials for LIBs, demonstrating a high initial storage capacity of 450 mA h^−1^ at 0.03 A g^−1^ but with a badly stable performance.^20^ After that, Ji *et al.* prepared composite CNFs from PAN/PPy (polypyrrole), exhibiting a large reversible storage capacity of 556 mA h g^−1^ at 0.05 A g^−1^ and superior stability.^21^ Although a various types of CNFs have been fabricated using different sources and also demonstrated excellent electrochemical performance in LIBs, while most of the CNFs are originated from non-renewable precursors consequently limiting its large-scale applications. Therefore, the importance of finding green and sustainable resources for manufacturing highly efficient performance CNFs materials and to reduce the consumption of fossil fuel resources are a pressing problem for almost each scholar.

Nature offers various types of biomass sources, which have been becoming more and more popular for production of low cost and high performance energy storage devices.^22–25^ Walnut, as one kind of lignocellulosic biomass, is widely grown and multifunctional plant in China with containing more than 70% cellulose and lignin, which have drawn great attention as a promising candidate for carbon precursor. In our earlier work, we have been demonstrated to effectively utilize this waste walnut shell to prepare the walnut shell-derived nanofibers *via* electrospinning for the first time. While in this work we further improved the liquefied ratios using more biomass resources and optimized the carbonization process. After one-step carbonization, the resultant CNFs with abundant pore structure and good flexibility were obtained, which directly use as binder-free electrode materials for LIBs. The effects of carbonization temperature on morphology and structure of electrospun nanofibers were characterized systemically, and the electrochemical performance was investigated intensively as anodes material for LIBs. The results demonstrate the great potential for converting the waste walnut shell to high-value electrode materials for energy storage.

## 2 Results and discussion

### 2.1 Characterizations of morphology and structure

**Fig. 1:**
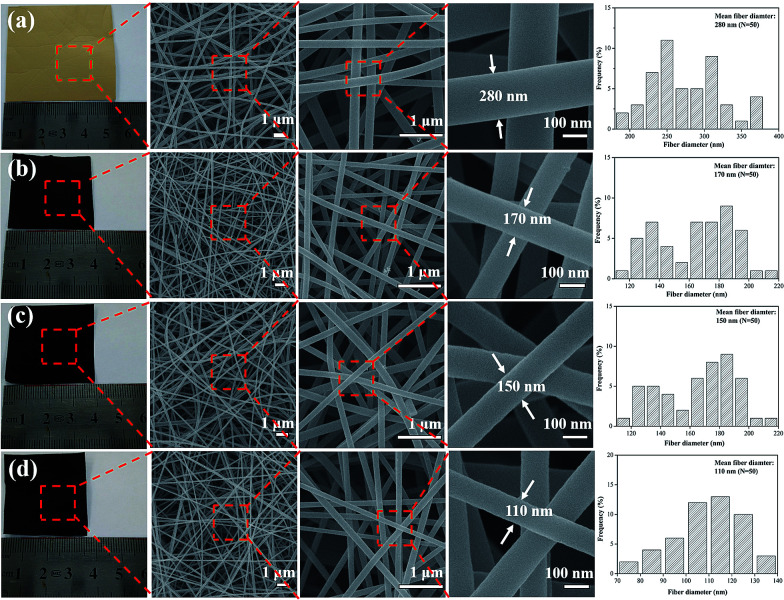
Photographs and FE-SEM images of electrospun nanofibers treated at different carbonization temperature: (a) as spun nanofibers, (b) 1000 °C, (c) 1500 °C, (d) 2000 °C.

In our previous work,^26^ we found that the resinificated solution (RS) is consist of low molecular weight compounds that lack of chain matrix or structural entanglements, thus a single RS at any concentration is a great challenge for electrospun continuous nanofibers. After by introducing PVA into the RS, the nanofibers start generation because of improved fluid properties and electrospinability. When the PVA content went 20% in RS, both the solution preparation and the electrospinning were easily. The morphology and microstructure of walnut shell-derived nanofibers and corresponding CNFs were observed by FE-SEM, as shown in Fig. 1. RS/PVA mixtures of 8 : 2 mass ratio, was readily electrospun and yielded bead-free nanofibers (Fig. 1a), and the electrospun nanofibers exhibit long and smooth surface morphology with diameter of 280 nm. After one-step carbonization, the resultant CNFs still maintained a fibrous geometry and integrity. When the carbonization temperature increasing from 1000, 1500, to 2000 °C, the diameter of CNFs decreases from 170, 150, to 110 nm because large amount of small molecules were released at high temperatures during carbonization (Fig. 1b–d). Furthermore, compared with the image size of electrospun nanofibrous mats, the carbon nanofibrous mats were only slightly decreased, which demonstrated higher yield of carbon and fairly large compared to carbon fibers produced with other precursors from biomass.^22,23^

**Fig. 2:**
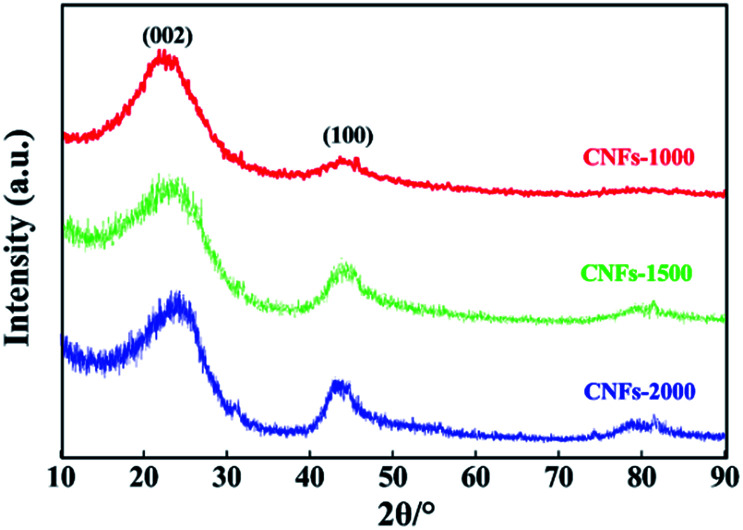
XRD patterns of walnut shell-derived CNFs-1000, CNFs-1500 and CNFs-2000.

XRD analysis and Raman spectroscopy were used to characterize the degree of graphitization of electrospun nanofibers during the carbonization process. As presented in Fig. 2, two broad reflections located at the 2*θ* ∼23° (major) and ∼44° (minor) were observed for each of the carbonized samples, which are assigned to typical crystallographic plane of (002) and (100) in graphitic carbon.^27^ The height and peak width at half height of the two peaks correlated with the degree of graphitization of the CNFs. Analysis of the (002) reflection gives the average interlayer spacing (*d*_(002)_, calculated by the Bragg equation) of 0.402, 0.395 and 0.377 nm with increasing carbonization temperatures from 1000, 1500 and 2000 °C, respectively. In addition, a broad band at about 43° (100) is apparently observed in CNFs-1500 and -2000 while weakly observed in CNFs-1000, which indicative disordered state, amorphous carbonaceous interlayers with a very low amount of crystalline phases at the low carbonization temperature and believed to be a results of the precursor used here follows a different mechanism for structure formation. Generally, an amorphous or poorly degree of molecular orientation in the precursor fibers will not develop large carbon crystallites.

**Fig. 3:**
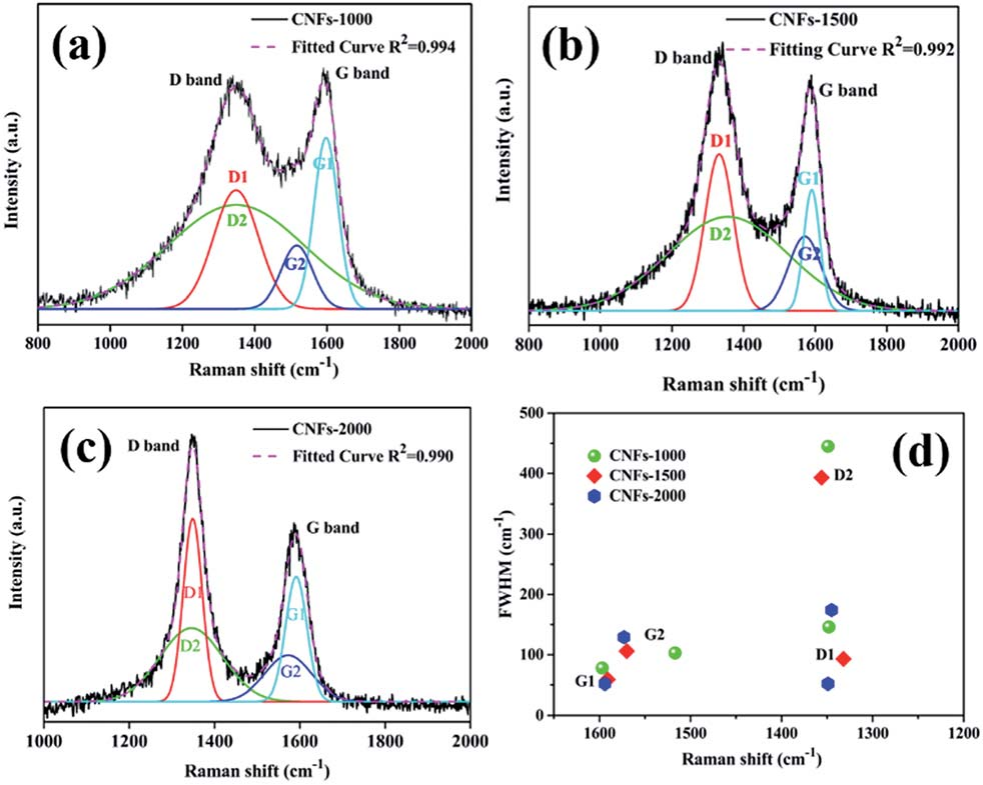
Raman spectra of walnut shell-derived CNFs-1000 (a), CNFs-1500 (b), CNFs-2000 (c) with fitting results by using Gaussians; (d) plots between Raman shift (*ν*) and FWHM (*Γ*) listed in Table 1, obtained by the peak deconvolution using Gaussians.

Raman spectroscopy was also used to characterize the graphite structure of CNFs. Fig. 3 presents the obvious structure of carbonaceous materials where two bands are observed. The strong peak at ∼1340 cm^−1^ is the defect or disordered sp^2^ carbon (D) band, while the peak at ∼1590 cm^−1^ is the E_2g_ graphite (G) band.^28^ The relative intensity ratio of “D-band” to “G-band” (*I*_D_/*I*_G_) indicates the graphite content of the carbonaceous materials. However, in this work, Gaussian fitting was used to deconstruct the D- and G-bands into four peaks (G1, D1, G2, and D2) to calculate the graphitic content (Fig. 3a–c). The G1/D1 peaks represent the small basal planes with a bond angle order, and the G2/D2 peaks represent amorphous sp^2^ carbon with bond angle disorder, respectively, following same deconvolution method proposed by Shimodaira and Masui.^29^ The basal planes correspond to the nanocrystalline graphite, consisting of liquefied walnut shell benzene rings and conjugated aromatic hydrocarbons after heating, while the sp^2^ carbon maybe originated from the non-aromatic hydrocarbons. Table 1 lists the detailed parameters of the deconvolution peaks.

**Table 1:** Fitting parameters in CNFs obtained from fitting four Gaussians, D1, D2, G1, and G2, to the Raman spectra

Sample	D1		D2		G1		G2		*I*_D1_/*I*_G1_	*I*_D2_/*I*_G2_	*I*_G2_/*I*_G1_
	*ν* (cm^−1^)	*Γ* (cm^−1^)	*ν* (cm^−1^)	*Γ* (cm^−1^)	*ν* (cm^−1^)	*Γ* (cm^−1^)	*ν* (cm^−1^)	*Γ* (cm^−1^)			
CNFs-1000	1348	146	1349	445	1597	78	1517	103	0.76	1.35	0.52
CNFs-1500	1332	93	1356	393	1591	59	1570	106	1.22	1.30	0.54
CNFs-2000	1349	52	1345	174	1594	52	1573	129	1.34	1.16	0.72

In order to prove which peaks present more significant variations in position and width, full width at half-maximum (FWHM) (*Γ*) *vs.* Raman shift (*ν*) is plotted in two dimensions in Fig. 3d. In general, the following features are observed: (1) the G1 peak position is very stable at 1594 ± 3 cm^−1^ with the FWHM of 65 ± 13 cm^−1^. (2) The D1 peak position is also stable at 1340 ± 8 cm^−1^ while its FWHM is larger and scattered (G1 widths decreased from 78 cm^−1^ in 1000 °C to 59 cm^−1^ in 1500 °C and to 52 cm^−1^ in 2000 °C, and D1 widths decreased from 146 cm^−1^ in 1000 °C to 93 cm^−1^ in 1500 °C and to 52 cm^−1^ in 2000 °C, respectively), indicating decreased bond disorder of the basal planes from high temperature. (3) With regard to the G2 and D2 peaks, both position and FWHM are widely distributed. Similar results were found by Shimodaira and co-workers,^29^ suggest that the increased formation of disordered sp^2^ carbons at a high-treatment temperature.

According to the Macedo and co-workers,^30^ a lowering intensity ratios *I*_D_/*I*_G_ of samples implied to increasing basal plane sizes (graphitic content). Similarly, the integrated intensity ratios *I*_D1_/*I*_G1_ and *I*_D2_/*I*_G2_ are calculated the nanocrystalline size along the basal plane. The *I*_D1_/*I*_G1_ ratio is generally referred as inversely proportional to the in-plane crystallite, which increases from 0.76 to 1.34 suggest that the basal plane size is estimated to decrease at higher carbonization temperature. While the *I*_D2_/*I*_G2_ ratio is regarded as proportional to the sp^2^ carbon cluster sizes, which decreased from 1.35 to 1.16 also suggest decreased amorphous sp^2^ carbon cluster sizes from higher carbonization temperature. Finally, the *I*_G2_/*I*_G1_ ratio, a reference value present the relative disordered to ordered contents, increased from 0.52 at 1000 °C to 0.54 at 1500 °C, and to 0.74 at 2000 °C. Meaning that the proportion of disordered domains became higher with increasing carbonization temperature.

**Fig. 4:**
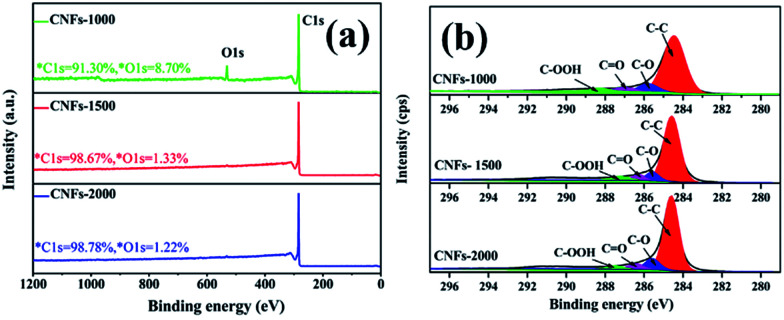
XPS spectrum of walnut shell-derived CNFs at different carbonization temperature: (a) survey; (b) C 1s.

After one-step carbonization, the surface elemental composition of carbonized samples was characterized by XPS measurement. As presented in Fig. 4a, there are two obvious peaks observed for C 1s and O 1s appear in the survey spectrum range of 1200–0 eV, confirming the presence of these atoms in the walnut shell-derived CNFs samples. The oxygen content decreased sharply from 8.70% to 1.22%, while the carbon content increased from 91.30% to 98.78% with increasing carbonization temperature, which revealed that more surface oxides suffered a great loss and abundant carbon was generated after higher temperature. Fig. 4b show the C 1s spectrum which was decomposed into four component peaks ranging from 280 to 296 eV according to our previous study.^31^Table 2 lists the detail parameters of the fits of the C 1s region. Graphite content (C–C) of the carbonized nanofibers gradually increases with increasing carbonization temperature. It is speculated that little heteroatoms in the matrix structure revealed and matrix structure rearrangement increased dramatically which resulted in carbon atoms more compacted and the partial unstable carbon generated graphene layers.

**Table 2:** Results of the fits of the C 1s region

Sample	Peak from C 1s spectrum binding energy			
	C–C	C–O	C=O	C–OOH
CNFs-1000	69.50%	16.10%	7.30%	7.10%
CNFs-1500	79.23%	11.09%	5.96%	3.37%
CNFs-2000	80.93%	8.25%	5.67%	5.06%

**Fig. 5:**
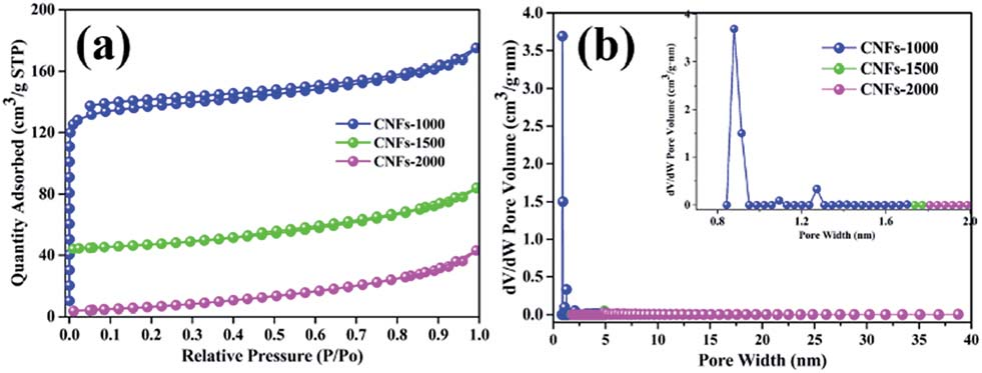
N_2_ adsorption/desorption isotherms (a) and pore size distribution (b) of nanofibers at different carbonization temperature.

Furthermore, the BET specific surface areas and the DFT pore size distribution of carbonized nanofibers were characterized using nitrogen adsorption/desorption analysis at −196 °C. As shown in Fig. 5a, the isotherm of the CNFs-1000 could be classified as type I under the basis of the Brunauer classification. The adsorption behavior sharply increased in low relative pressure and approached a plateau at high relative pressure, which can be ascribed to N_2_ adsorption in more micropores.^32^ However, at higher carbonization temperature, CNFs-1500 and CNFs-2000 exhibited type II N_2_ isotherms, typical of non-porous and macroporous materials with weak affinities to nitrogen. Table 3 lists the surface area and microporosity of carbonized nanofibers. From Table 3, the CNFs-1000 shows the largest BET surface area of 420 m^2^ g^−1^ which could be explained by volatilization of degradation by-products. As temperature increased to 1500 °C, specific surface area sharply decreased to 39 m^2^ g^−1^. As further increased to 2000 °C, specific surface area decreased to only 7 m^2^ g^−1^. The relatively lower surface area could be ascribed to the thermal treatment which resulted in structural rearrangement and thus less microporosity at higher temperature.^33^ Similarly, the pore volume of CNFs-1000 shows the highest. While carbonization temperature increased to 1500 or 2000 °C, the velocity of this process increased dramatically, thus leading to the dramatic decrease in pore volume. Conversely, the average pore width of CNFs increases from 2.4 nm to 8.2 nm with increasing carbonization temperature from 1000 °C to 2000 °C, it seems meaningless because of its very small specific surface area.

**Table 3:** Porous structure parameters for walnut shell-derived CNFs

Sample	*S*_BET_ a	*V*_tot_ b	APW^c^	IDC^d^	ICC^e^	ICE^f^
CNFs-1000	420	0.257	2.4	341.3	198.3	58.1
CNFs-1500	39	0.057	7.9	182.5	107.7	60.4
CNFs-2000	7	0.031	8.2	109.1	68.9	63.2

a *S*_BET_: BET method.

b *V*_total_: total pore volume measured at *P*/*P*_0_ = 0.99.

c APW: adsorption average pore width (4*V*/*A* by BET).

d The initial discharge capacity.

e The initial charge capacity.

f The initial coulombic efficiency.

### 2.2 Electrochemical performance

**Fig. 6:**
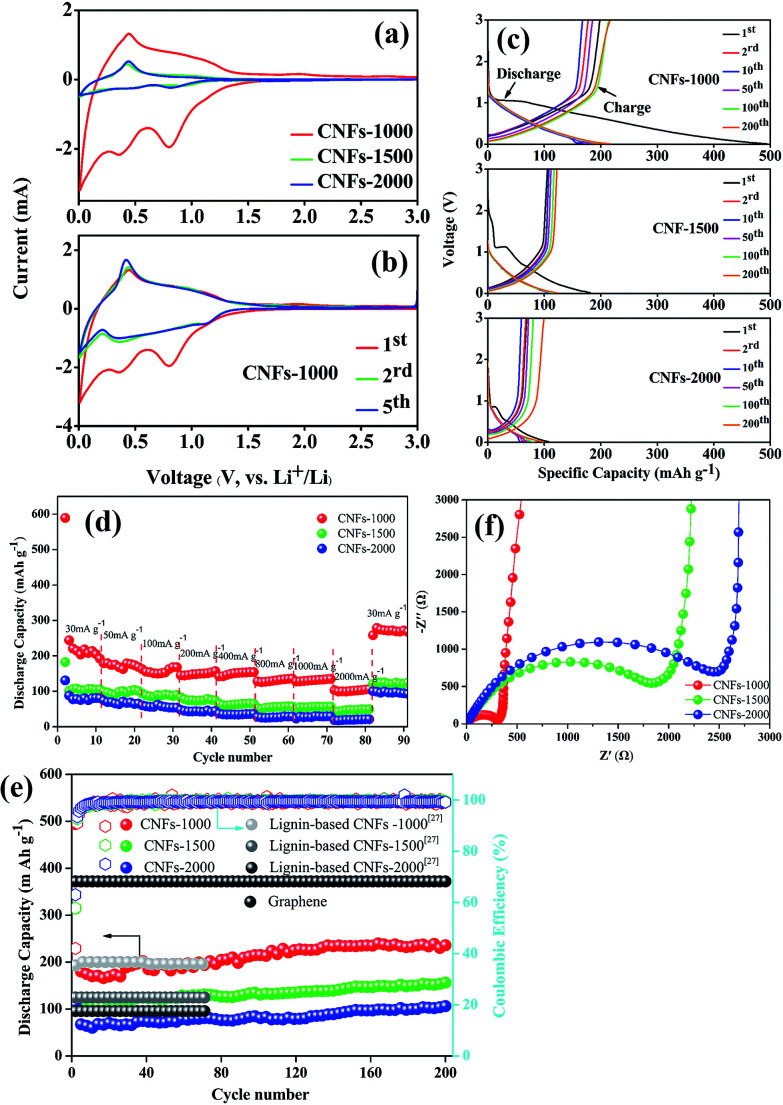
Electrochemical performance of LIBs. CV curves of walnut shell-derived CNFs at a scan rate of 0.5 mV s^−1^ between 0.01–3.0 V at first cycle (a), and CNFs-1000 at first five cycles (b); (c) charge/discharge curves of CNFs-1000, CNFs-1500, and CNFs-2000 at current density of 100 mA g^−1^; (d) discharge capacities of walnut shell-derived CNFs at different current rates; (e) cyclic performances and corresponding coulombic efficiency of walnut shell-derived CNFs at a current density of 100 mA g^−1^; (f) EIS of walnut shell-derived CNFs.

All the carbonized nanofibers were assembled into Li-ion batteries (2025-type coin cells) so as to compare the performance of these materials as a function of the preparation conditions. Fig. 6a shows the CV curves of carbonized nanofibers electrodes at first cycle in Li-ion electrolyte, where the shape of curves reflected the electrochemical reaction kinetics of Li-ion insertion and deinsertion. In the insertion process, there is one small oxidation peak located at ∼0.5 V, corresponding to the transformation of Li-ion insertion into nanopores on the surface of fibrous mats.^34^ In the deinsertion process, there is two irreversible reductive peaks around at ∼0.4 and ∼0.8 V, attributing to the electrolyte decomposition reaction from the fibrous mats and the formation of a solid electrolyte interphase (SEI).^35^ The shape of redox peaks in following CV curve was almost coincident (Fig. 6b), implying the irreversible capacity mainly occurs in the first cycle and the CNFs electrodes exhibit a stable Li-ion insertion and deinsertion mechanism.

Fig. 6c presents the charge–discharge curves of the electrodes (CNFs-1000, CNFs-1500, CNFs-2000) for the 1st, 2nd, 10th, 50th, 100th, 200th cycle at 100 mA g^−1^ with a potential window of 0.01–3.0 V. In the first discharge curves, three electrodes present a clear plateau around at ∼1.2 V, which is mainly attributed to the SEI film rapid generation caused by the electrolyte decomposition. Meanwhile, high-treatment temperatures have a more significant influence on the electrochemical performance for carbonized samples. Compared with the CNFs-1000 electrode which has a superior initial discharge capacity of 341.5 mA g^−1^, the CNFs-1500 (or 2000) electrode exhibits an inferior capacity of 182.5 mA g^−1^ (or 107.7 mA g^−1^). This dramatic difference in discharge capacity between 1000 and 1500 °C (or 2000 °C) ascribed to the different microstructure (such as BET surface areas, porous structure, and interlayer spacing) and graphite-like crystallite layers, which are easy for lithium ions insertion and deinsertion.^36^ For each of sample, the sloping capacities also slightly decrease with increase of carbonization temperature, while this phenomenon likely attributed to a decreasing position of intercalation (such as basal plane, edges, and the interactions between lithium and heteroatoms) and a lowering of interlayer spacing. Table 3 summarizes the corresponding parameters of three carbonized nanofibers at first cycle. As a result, the high irreversible capacity between initial discharge and charge may be attributed to the SEI film rapid generation. Whereas with carbonization temperature increases, the initial coulombic efficiency increases from 58.1% to 63.2%, originating from the sufficient to eliminate residual heteroatom contents or organic functionalities, which play a more important role to reduce irreversible Li-ion insertion reaction. In subsequent cycles, there is no discernible plateau for each of sample, indicative of the disordered structure observed where Li-ion insertion sites into the CNFs are electronically and geometrically distinct.^37,38^ This lack of plateau is also observed in electrospun CNFs from other precursors where long range crystalline order is absent.^39–41^ For CNFs-1000 electrode, there is still 200 mA h g^−1^ specific capacity after 200 cycles, implying excellent repeatability and stable Li-ion insertion/deinsertion stability.

The rate performance comparison of the electrode under different carbonization temperature was also investigated. As shown in Fig. 6d, the CNFs-1000 electrode exhibits the highest rate capacities at each current density from 30 to 2000 mA g^−1^, and when the current density is returned to 30 mA g^−1^, the specific capacity increases to 271.7 mA h g^−1^ with stable cycling. For disordered carbons, the higher the carbonization temperature achieved, the lower the capacity obtained, which may be related with the sharp decrease of porosity, and the relatively low graphitic content. This phenomenon is consistent with previous studies of disordered carbon.^42,43^ Meanwhile, there is an interesting observation that when the current density is turned back to 30 mA g^−1^, the specific capacities of all CNFs electrodes are much higher than the original value. The improvement of the rate performance is attributed to the abundant porosity on the surface of CNFs, which reduces the Li-ion insertion and deinsertion distances, and insertion process likely porosity filling is also an important Li-ion storage mechanism for carbonized electrode especially cycled at high current density.^34^ This phenomenon is more obvious in long cycling performance.

Fig. 6e compares the long cycle tested of the different carbonized CNFs investigated at 100 mA g^−1^. We can see that the CNFs-1000 electrode maintains a stable discharge capacity of 239.6 mA h g^−1^ after 200 cycles. However, for CNFs-1500 and -2000 electrode, the capacities are only 122.7 and 99.37 mA h g^−1^, respectively. The reason for the specific capacities sharply decrease at higher carbonization temperature has been discussed above. The initial capacities of three CNFs electrodes show an obvious capacity decrease during the first ten cycles, attributed to the SEI film formation. In the subsequent cycles, the capacities of each electrode improved nearly 20% compared with the initial values after 200 cycles with coulombic efficiencies of nearly 100%. This dramatic increase in capacitance can be explained in a layer of gelatinous material that forms on the surface of the CNFs electrode, which can provide additional capacity, or the original SEI film cracks to generate a new thinner SEI film which leads to an increased specific capacity.^44,45^ However, due to the bad electric conductivity, the capacity of CNFs is far less than that of graphene, but is comparable with that of electrospun carbon nanofibers from lignin.^29^

To further confirm the difference electrochemical performance, the carbonized electrodes were also examined by electrochemical impedance spectrometry (EIS), shown as Fig. 6f. All of the Nyquist plots consist of a single depressed semicircle in the high-to-medium frequency region and an inclined line in the middle-to-low frequency region, which can be attributed to the charge-transfer resistance (*R*_ct_) and a Li-ion diffusion process.^46^ Compared to the different carbonized electrodes, the CNFs-1000 electrode exhibits a lowering interfacial resistance and charge transfer resistance, which may be attributed to its more graphite-like crystallite layers and abundant BET surface area, pore volume. On the one hand, the more graphite-like crystallite layers will be conducive to the reversible insertion and deinsertion of lithium-ion. On the other hand, the large surface area and pore volume can provide a sufficient electrode–electrolyte interface to enhance the reactivity and facilitate lithium-ion transport in the solid phase, thus indicating its excellent electrochemical performance.

**Fig. 7:**
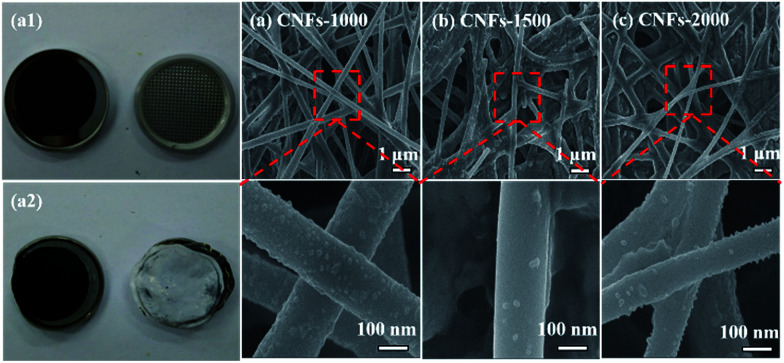
Photographs of CNFs-1000 electrode before (a1) and after (a2) cycled and FE-SEM images of three CNFs electrodes after 200 galvanostatic charge–discharge cycles at a current density of 100 mA h g^−1^: (a) CNFs-1000, (b) CNFs-1500, (c) CNFs-2000.

In order to verify the mechanism for the good cycling stability of carbonized samples, we opened the cell and measured the structure changes in morphology by FE-SEM after 200 cycles at 100 mA h g^−1^. Compared with the photograph of CNFs-1000 electrode before and after cycles, the original shape and size of the electrode has hardly changed, proving an excellent structure stability, as shown in Fig. 7a1 and a2. Fig. 7a–c lists the FE-SEM characterization of the cycled electrodes. It is obvious shown that three cycled electrodes still maintain structure integrity and fibrous geometry with similar diameters and without obvious fracture and pulverization compared to that in Fig. 1, showing better the good cycling performance. However, a slightly rough surface is observed in each of the cycled electrode due to the SEI film generation on the surface of CNFs that can be serve as protective layer to improve the cycle performance.^47^ The excellent electrochemical performance demonstrates great potential for walnut shell-derived CNFs to applications in LIBs.

## 3 Experimental

### 3.1 Materials

The walnut shell was from Yunnan Province, China. Phenol (C_6_H_5_OH), sulfuric acid (H_2_SO_4_, 98 wt%), formaldehyde (CH_2_O, 37 wt%), sodium hydroxide (NaOH) and polyvinyl alcohol (PVA, CAS number: 8068-05-1) was purchased from Aladdin Industrial Corporation. All chemicals were used as received without further purification.

### 3.2 Synthesis of walnut shell-derived carbon nanofibers (CNFs)

In our previous work,^26^ electrospinning solution was synthesized in two steps. First, walnut shell was liquefied into small molecules by liquefaction technique used phenol as liquefying auxiliary (this work mass ratio of walnut shell/phenol/H_2_SO_4_ = 1/3/0.03, liquefied at 150 °C for 2 h). Second, the mixture of liquefied walnut shell, formaldehyde and sodium hydroxide at a molar ratio of 1/1.45/0.2 were loaded into a three-neck flask followed by resinification technique to obtained resinificated solution (RS). In order to obtained bread-free electrospun nanofibers, 12 wt% PVA solution added into RS for stirring 2 h to adjust the electrospinability.

The homogenous electrospinning solution was placed in a 5 mL disposable syringe with a 23 gauge needle and was electrospun using an electrospinning device (ET-2535H, Ucalery, China). During working, the needle was connected to the positive pole of a voltage generator, and a roller of 30 cm diameter covered with an aluminium foil and connected to the negative pole of the power supply (ground) for collecting the nanofibers. The spinning was performed at 16 kV with a flow rate of 0.045 mL min^−1^, and the spinning distance was 35 cm between the tip of needle and the roller. The collected electrospun mats was dried at room temperature and peeled off from the aluminum foil. The dried mats were then carbonized in the range of 1000, 1500 and 2000 °C at 5 °C min^−1^ and kept at current temperature for 1 h under a flow of argon atmosphere, denoted as CNFs-1000, CNFs-1500 and CNFs-2000.

### 3.3 Characterization

Several characterization tools were used to characterize the CNFs. Morphologies of nanofibrous mats were investigated with JSM-7500F field emission scanning electron microscopy and the diameter distribution of each sample was obtained from 50 randomly selected nanofibers that were image-analyzed (ImageJ software). The structure of carbonized nanofibers was characterized by X-ray diffraction patterns (XRD, Philips, Netherlands) using Cu-Kα radiation (wavelength *λ* = 1.54 Å) and Raman spectra (Raman-Station 400, PerkinElmer, MA, USA). Chemical surface composition of carbonized samples was characterized by X-ray photoelectron spectroscopy (XPS, ESCALAB 250Xi, Thermo Fisher, USA) with a monochromated Al Kα X-ray source of 1486.6 eV. The Brunauer–Emmett–Teller (BET) specific surface areas and the density functional theory (DFT) pore size distribution of carbonized samples were determined using N_2_ adsorption–desorption (ASAP 2020 Plus HD88, Micromeritics, USA) at −196 °C after outgassed at 300 °C for 6 h under vacuum.

### 3.4 Electrochemical test

The flexible carbonized mats are cut into circular disks with a diameter of 16 mm and directly used as the electrode without binder or conductive additive. The mass of each disk is about ∼3.0 mg. The electrochemical performance was tested in 2025-type stainless steel coin cells with Celgard 2500 as the separator, and lithium metal as the counter electrode. The electrolyte was 1.0 M LiPF_6_ dissolved in a 1 : 1 volumetric solution of ethylene carbonate and dimethyl carbonate (Tianci Co. Ltd). Cyclic voltammetry (CV) tests and electrochemical impedance spectroscopy (EIS) measurements were performed on an electrochemical workstation (CHI760 E, Chenhua, China). The galvanostatic discharge–charge tests were conducted using a Multi-channel Lanhe Battery Measurement System (Lanhe, China) with a potential window of 0.01–3.0 V.

## 4 Conclusions

In summary, we have demonstrated a novel method to use of sustainable lignocellulosic biomass—walnut shell as new carbon-derived precursors for the controlled synthesis of mechanically flexible and free-standing CNFs mats with abundant pore structure as electrode materials for Li-ion batteries. Carbonization of CNFs mats at high temperature resulted in lower specific surface area and graphite-like layer, which are unfavorable for storing lithium ion that resulted in lower electrochemical performance. As a consequence, the fibrous mats carbonized at 1000 °C have the best overall performance, exhibiting high specific capacity with 271.7 mA h g^−1^ at 30 mA g^−1^, extraordinary rate capability with 131.3 mA h g^−1^ at 1 A g^−1^ and excellent cycling stability without any decay after 200 cycles at 100 mA h g^−1^ in Li-ion batteries. This finding demonstrates the great potential for converting the low-cost biomass to high value carbon materials for applications in energy storage.

## Conflicts of interest

There are no conflicts to declare.

## Supplementary Material
